# Altered profile of immune regulatory cells in the peripheral blood of lymphoma patients

**DOI:** 10.1186/s12885-019-5529-0

**Published:** 2019-04-05

**Authors:** R-M Amini, G. Enblad, P. Hollander, S. Laszlo, E. Eriksson, K. Ayoola Gustafsson, A. Loskog, I. Thörn

**Affiliations:** 1 Clinical and Experimental Pathology, Department of Immunology, Genetics and Pathology, Uppsala University and Uppsala University Hospital, Rudbeck Laboratory, C5, SE-75185 Uppsala, Sweden; 2 Experimental and Clinical Oncology, Department of Immunology, Genetics and Pathology, Uppsala University, Uppsala, Sweden; 3 Clinical Immunology, Department of Immunology, Genetics and Pathology, Uppsala University, Uppsala, Sweden; 4 Medical Genetics and Genomics, Department of Immunology, Genetics and Pathology, Uppsala University, Uppsala, Sweden; 5 Lokon Pharma, AB, Uppsala, Sweden

**Keywords:** Immune regulatory cells, NK cells, T cells, Myeloid derived suppressor cells, High-grade B cell lymphoma, Hodgkin lymphoma

## Abstract

**Background:**

Regulatory immune cells may modulate the lymphoma microenvironment and are of great interest due to the increasing prevalence of treatment with immunotherapies in lymphoma patients. The aim was to explore the composition of different immune regulatory cell subsets in the peripheral blood of newly diagnosed lymphoma patients in relation to treatment outcome.

**Methods:**

Forty-three newly diagnosed patients with lymphoma were included in the study; 24 with high-grade B-cell lymphoma (HGBCL) and 19 with classical Hodgkin lymphoma (cHL). Peripheral blood was prospectively collected and immune regulatory cells were identified by multi-color flow cytometry and analyzed in relation to healthy blood donors and clinical characteristics and outcome.

**Results:**

The percentage of CD3-positive T-cells was lower (*p* = 0.03) in the peripheral blood of lymphoma patients at diagnosis compared to healthy blood donors regardless of lymphoma subtype, although statistically, neither the percentage of monocytes (*p* = 0.2) nor the T-cell/monocyte ratio (*p* = 0.055) differed significantly. A significant decrease in the percentage of a subset of regulatory NK cells (CD7^+^/CD3^−^/CD56^bright^/CD16^dim/−^) was identified in the peripheral blood of lymphoma patients compared to healthy blood donors (*p* = 0.003). Lymphoma patients also had more granulocytic myeloid-derived suppressor cells (MDSCs) (p = 0.003) compared to healthy blood donors, whereas monocytic MDSCs did not differ significantly (*p* = 0.07). A superior disease-free survival was observed for cHL patients who had an increase in the percentage of granulocytic MDSCs (*p* = 0.04).

**Conclusions:**

An altered profile of immune cells in the peripheral blood with a decrease in T-cells and regulatory NK-cells was observed in newly diagnosed lymphoma patients. CHL patients with higher percentages of regulatory NK cells and higher percentages of granulocytic MDSCs might have a better outcome, although the number of patients was low.

**Electronic supplementary material:**

The online version of this article (10.1186/s12885-019-5529-0) contains supplementary material, which is available to authorized users.

## Background

Immune suppression has long been associated with classical Hodgkin lymphoma (cHL) where severe lymphopenia is included in the International Prognostic Score (IPS) [1] probably reflecting impaired host immunity, and an increase in activated (CD4^+^/CD25^+^) T-cells has been observed in the peripheral blood of long-term survivors of cHL [2]. Previous studies have shown that cHL patients with a decreased number of lymphocytes and increased monocytes, measured as a ratio between lymphocytes and monocytes in the peripheral blood at diagnosis have a worse clinical outcome [3, 4]. Similar results, with an inferior outcome for patients with a decreased lymphocyte/monocyte ratio have also been shown for patients with other lymphoma types, such as diffuse large B-cell lymphoma (DLBCL) and follicular lymphomas [5, 6]. T regulatory cells play an important role in self-tolerance and autoimmunity and the role of T regulatory cells in different malignant disorders has been diverse [7]. In B-cell lymphoma patients, an increase of T regulatory cells was observed at diagnosis and a decrease after therapy [8]. In cHL, a correlation with poor prognostic markers was observed [9] but the role of T regulatory cells in lymphoma patients is not fully elucidated.

Immune cells in the peripheral blood of lymphoma patients have been subject to attention lately due to the possible relation to tumor microenvironment and immune status. Important immune regulatory mechanisms need to be better defined in order to be targeted with immunotherapies like those directed towards the programmed death receptor 1 (PD-1) immune checkpoint pathway. PD-1 is a major inhibitory receptor on effector T-cells and T-cells with high PD-1 expression have reduced ability to eliminate tumor cells [10]. Targeting the PD-1/PD-Ll interactions influences not only T-cells, but also NK cells, monocytes and myeloid-derived suppressor cells (MDSCs) and it might be important to evaluate all these cell populations in order to identify patients who might respond favorably to PD-1 pathway inhibitors [11].

NK cells are bone-marrow derived lymphoid cells and part of the innate immune system, characterized by the expression of CD56 and/or CD16 [12]. A subset of regulatory NK cells has been identified that express CD7^+^/CD3^−^/CD56^bright^ /CD16^dim/−^ in contrast to NK cells that are CD7^+^/CD3^−^/CD56^+^/CD16^+^. These regulatory NK cells may after activation become cytotoxic towards autologous CD4^+^ T cells [13]. In other studies on patients diagnosed with cancer in the head-neck [14] and breast [15], a decrease in the peripheral blood of regulatory NK cells has been observed. How these cells appear in lymphoma patients has previously not been well investigated, although one recently published study has shown expansion of PD-1 expressing regulatory NK cells in lymphoma patients [11].

MDSCs are myeloid cells with the ability to suppress other immune cells. MDSCs comprise a heterogeneous population of myeloid cells and there are two major types of MDSCs; granulocytic and monocytic derived [16–18]. Granulocytic MDSCs are most commonly defined as CD14 negative and CD11b expressing cells that express the common myeloid marker CD33 but lack the expression of HLA-DR [17, 19], whereas monocytic MDSCs express CD14. The granulocytic MDSCs share the same immunophenotype as polymorphonuclear (PMN) granulocytic cells but will be different regarding density and can thus be separated by density gradient centrifugation of peripheral blood mononuclear cells (PBMC) [17, 18].

It remains unclear how different immune cells interfere and contribute to the pathogenesis of lymphoma development, and this needs to be further explored since immunotherapies are becoming increasingly important as treatment options [20].

The aim of this study was to investigate different subsets of immune regulatory cells in the peripheral blood at lymphoma diagnosis by the use of a multicolor flow cytometry approach to identify immune regulatory cells in relation to clinical characteristics and treatment outcome.

## Methods

### Patients

In total, 43 patients were included in the study. Patients were recruited from the U-CAN consortium of prospectively collected tumor and blood samples at diagnosis and diagnosed during 2013–2016 [21]. All clinical information was retrieved from patient records. All patients were treated according to National treatment guidelines.

The study was approved by the Regional Ethical Committee (Dnr 2010/198 and Dnr 2013/059). Peripheral blood samples from 15 healthy blood donors were included as controls (mean age 44, range 20–69) with male to female ratio 3:1.

Twenty-four patients were diagnosed with high-grade B-cell lymphoma (HGBCL) most often DLBCL but in some cases the diagnosis was made on core needle biopsies only and a correct subclassification according to the WHO classification [22] was thus not possible to perform. Nineteen patients were diagnosed with cHL. All diagnoses were reevaluated and for all patients peripheral blood in heparinized tubes at diagnosis was collected and available for multi-color flow cytometry analyses. Ficoll Isopaque-gradient separation of mononuclear cells was done according to manufacturer instructions prior to freezing the cells in DMSO in liquid nitrogen before analyses. The different cell types investigated are summarized in Table 1. Gating strategies of the different cell populations are presented in Additional file 1.

**Table 1:** Percentages of the different immune cells of viable peripheral blood mononuclear cells in patients with classical Hodgkin lymphoma (cHL), high-grade B-cell lymphoma (HGBCL) and healthy blood donors. Mann-Whitney U test was performed comparing the different groups and *p*-values refer to all lymphoma patients vs healthy blood donors

Cell type	CHL	HGBCL	Blood donor	*p*-value
	Mean/Median (range) std	Mean/Median (range) std	Mean/Median (range) std	
T-cells CD3+	41.8/45.1 (3.9–65) 15.6	40.2/37.9 (14.8–61.4) 11.8	50.7/53.4 (36–64.1) 9.5	0.03
T-regulatory cells CD3^+^/CD4^+^/CD25^+^/CD127^low^	4.4/3.8 (0.4–10.9) 2.4	5.3/4 (1.2–30.5) 5.9	4.1/3.4 (0.4–8) 2.5	0.6
CD4+/CD8+ ratio	2/1.6 (0.18–4.9) 1.4	2.9/1.9 (0.5–16.2) 3.4	2/1.6 (0.53–7.4)	0.7
Monocytes CD33^++^/CD14^+^/HLA-DR^+^	20.9/20.6 (4–47) 11.7	25.2/22.8 (9.3–66) 11.8	18.7/18.5 (3.5–33) 8.8	0.2
NK cells CD7^+^/CD3^−^CD56^+^/CD16^+^	6.5/4.4 (0.29–27.6) 6.1	6.4/5.2 (0.2–24.8) 5.5	9/8.9 (0.04–19.4) 4.9	0.1
NK regulatory cells CD7^+^/CD3^−^/CD56^++^/CD16^dim/−^	0.28/0.21 (0.007–0.89) 0.26	0.31/0.25 (0.003–0.92) 0.25	0.54/0.44 (0.02–1.6) 0.36	0.003
Monocytic MDSC CD11b^+^/HLA-DR^−^/CD33^+^/CD14^+^	1.1/0.53 (0.043–3.7) 1.1	0.61/0.41 (0.024–2.2) 0.57	0.49/0.31 (0.015–2.7) 0.66	0.07
Granulocytic MDSC CD11b^+^/HLA-DR^dim^/CD33^dim^/CD14-	12.7/8.1 (0.6–83.8) 18.3	5.7/4.2 (0.4–19.3) 4.96	3.8/1.1 (0.18–17.4) 5.2	0.003

Abbreviations: *CHL* classic Hodgkin lymphoma, *HGBCL* high grade B-cell lymphoma, *std.* standard deviation, *MDSC* myeloid derived suppressor cells

### Flow cytometry

Three different 10-color tubes were used to identify the different cell subtypes. All samples were analyzed on the Navios instrument (Beckman-Coulter). Kaluza Analysis Software 1.2 (Beckman Coulter) and Infinicyte 1.7 flow cytometry software were used for data analysis. Approximately 0.5 × 10^6^ cells/tube were labeled with three different mixes of fluorescent labeled antibodies. All surface antigens were labeled for 10 min. in the dark at 20 °C. The cells in tubes without intracellular staining were washed once and then run immediately in the Navios instrument*.* For intracellular staining (e.g. FOXP3) the T-reg Detection Kit (Miltenyi Biotec) was used with fixation and permeabilisation according to manufacturer instructions. The following antibodies were used: PD-1- FITC (CD279, clone MIH4, Becton Dickinson), CD25-PE (clone 4E3, Miltenyi Biotec), CD45RA-ECD (clone 2H4LDH11LDB9 (2H4), Beckman Coulter), CD8-PC5.5 (clone SFCI21THy2D3, Beckman Coulter), CCR7-PC7(CD197 clone G043H7, Beckman Coulter), FOXP3-APC (clone 3G3,Miltenyi Biotec), CD127-APC-700 (clone R34.34, Beckman Coulter), CD3-APC-750 (clone UCHT1, Beckman Coulter), CD4-BV421 (clone RPA-T4, Becton Dickinson), CD45-KrO (clone J33, Beckman Coulter), CD161-FITC (clone 191B8, Miltenyi Biotec), Vα24Jα18-PE (clone 6B11, BioLegend), CD56-ECD (clone NKH-1, Beckman Coulter), CD7-PC7 (clone 8H8, Beckman Coulter), CCR7-APC (clone G043H7, BioLegend), CD16-APC-700 (clone 3G8, Beckman Coulter), CD13-FITC (clone SJ1D1, Beckman Coulter), CD115-PE (clone 9-4D2-1E4, BioLegend), CD14-ECD (clone RM052, Beckman Coulter), CD33-PC5.5 (clone D3HL60.251, Beckman Coulter), HLA-DR-PC7 (clone Immu357, Beckman Coulter), CD163-APC (clone GHI/61, BioLegend), CD11b-APC-750 (clone Bear1, Beckman Coulter), CD15-PB (clone 80H5, Beckman Coulter).

In this paper, we did, however, not include the following markers FOXP3, CCR7, CD45RA, PD1, CD115, CD163, CD161, Vα24Jα18.

### Statistical analyses

Disease-free survival (DFS) was calculated from the date of diagnosis to date of relapse or death as a result of lymphoma. Patients who died from a cause other than lymphoma and who were in remission were censored. Disease-free patients were followed from diagnosis to date of last follow-up. Patients who never achieved remission had a DFS of zero months. Survival curves and univariate analysis were performed using the Kaplan-Meier method, and the log-rank test was used to compare differences between groups. Appropriate cutoff values were determined by receiver operating characteristic curves calculated for each marker (Additional file 2). In addition, the median and mean values of the different cell populations for the healthy blood donors were also tested as cutoff values for survival analyses. The Mann Whitney U test and paired T-test were used to assign differences between the groups. A *p*-value < 0.05 was considered to be statistically significant. Statistical analyses were performed using Statistica 13 software (StatSoft, Scandinavia AB) and R 3.4.4 software (www.r-project.org).

## Results

The clinical characteristics of all patients are presented in Table 2. For the HGBCL patients, the male to female ratio was 2:1 and for the cHL patients it was 2.2:1.

**Table 2:** Clinical characteristics of all patients

	Hodgkin lymphoma *N* (%)	High-grade B-cell lymphoma *N* (%)	All lymphoma patients *N* (%)	Healthy blood donors *N* (%)
Patients	19	24	43	15
Male	13 (68)	16 (67)	29 (67)	9 (75)
Female	6 (32)	8 (33)	14 (33)	3 (25)
Unknown				3
Age				
Mean (range)	47 (22–84)	64 (35–85)	57 (22–85)	44 (20–69)
Relapse	1 (5)	1 (4)		
RT only		2 (8)		
R-CHOP		20 (83)		
ABV/ABVD	14 (74)			
BEACOPP	2 (10)			
Other treatment	3 (16)	2(8)		
PD	3 (16)	2 (8)		
DWD	3 (16)	3 (12)	6 (14)	
AWD	1 (5)	0 (0)		
Median follow-up time	23	18	19.8	
Mean follow-up time (range)	20 (1–40)	23 (3–52)	21 (1–52)	
ADF	15 (79)	19 (79)		
Stage				
1A	2 (10.5)	9 (38)		
1B	2 (10.5)	–		
2A	4 (21)	2 (8)		
2B	2 (10.5)	2 (8)		
3A	3 (16)	5 (21)		
3B	3 (16)	1 (4)		
4A	2 (10.5)	1 (4)		
4B	1 (5)	4 (17)		
B-symptoms	8 (42)	8 (33)	16 (37)	
IPS				
0–1	4 (21)	–		
2	7 (37)	–		
3	7 (37)	–		
> 4	1 (5)	–		
IPI				
0–1	–	12 (50)		
2	–	5 (21)		
3	–	4 (17)		
4	–	3 (12)		

Abbreviations: *RT* radiotherapy, *R-CHOP* rituximab, cyclophosphamide, doxorubicine, vincristine, prednisone, *ABVD* doxorubicine, bleomycin, vinblastine, dacarbazine, *AVD* doxorubicine, vinblastine, dacarbazine, *BEACOPP* bleomycin, etoposide, doxorubicin, cyclophosphamide, vincristine, procarbazine, prednisone

**Fig. 1:**
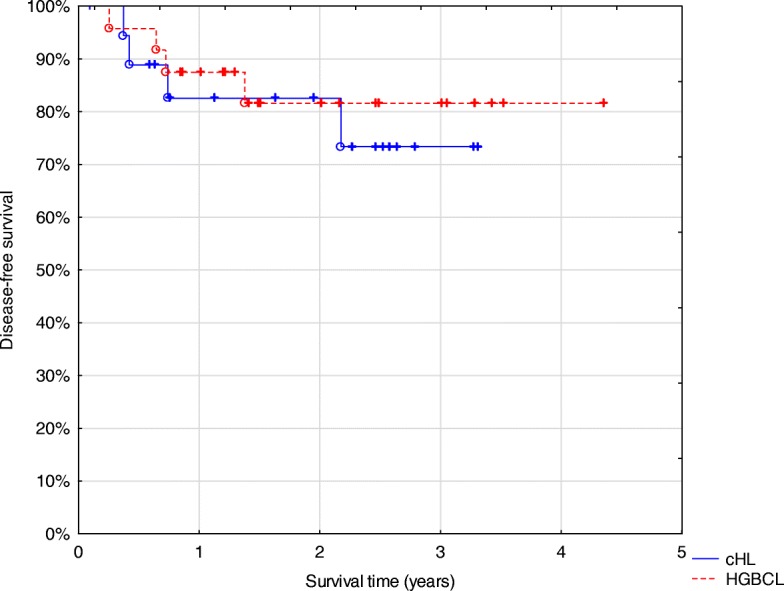
Disease-free survival of all patients included in the study. Abbreviations: *cHL* classical Hodgkin lymphoma (*n* = 19). *HGBCL* high grade B-cell lymphoma (*n* = 24)

DFS for all patients is presented in Fig. 1. The three-year DFS was 73% for cHL patients and 82% for those with HGBCL with no statistically significant survival differences.

**Fig. 2:**
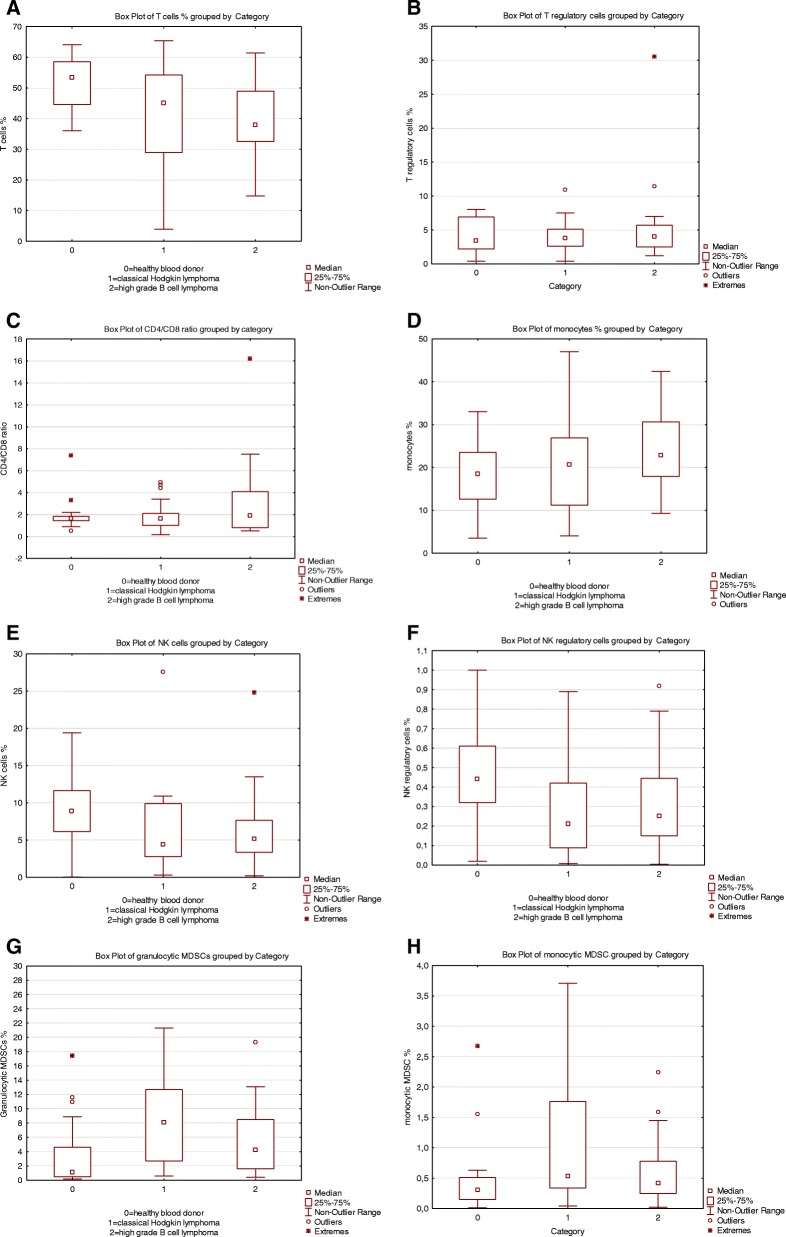
Boxplots of distributions of the different immune cells in percentage of viable peripheral blood mononuclear cells for each category and CD4/CD8 ratio: 0 = healthy blood donors, 1 = patients with classical Hodgkin lymphoma and 2 = patients with high-grade B cell lymphoma. **a**. = T cells *p*-value = 0.03, **b** = T regulatory cells p-value =0.6, **c** = CD4/CD8 ratio *p*-value = 0.7, **d** = Monocytes *p*-value = 0.2, **e** = NK cells *p*-value = 0.1, **f** = NK regulatory cells *p*-value = 0.003, **g** = granulocytic MDSCs *p*-value = 0.003, **h** = monocytic MDSCs *p*-value = 0.08

Comparisons between the different cell populations are presented in Fig. 2 a-h. The percentages of the different cell types and the CD4/CD8 ratio were calculated on all viable peripheral blood mononuclear cells and the percentages of positive cells are presented in Table 1.

A decreased percentage of CD3^+^ T cells was observed in lymphoma patients (*p* = 0.03) compared to controls. A tendency for a lower T cell/monocyte ratio was observed in lymphoma patients (*p* = 0.055) compared to controls (data not shown). T regulatory cells defined as CD3^+^/CD4^+^/CD25^+^/CD127^low^ (*p* = 0.6) did not differ significantly between lymphoma patients and controls. There was a significant difference in the percentages of regulatory NK cells defined as CD7^+^/CD3^−^/CD56^bright^/CD16^dim/−^ (*p* = 0.003) between lymphoma patients and controls where the lower percentages of these cells were detected in lymphoma patients. For NK cells (CD7^+^/CD3^−^/CD56^+^/CD16 ^+^) there was no significant difference (*p* = 0.1) between lymphoma patients and controls. Granulocytic MDSCs were significantly higher in the lymphoma patients (*p* = 0.003) compared to the controls, whereas percentages of monocytic MDSCs did not differ significantly between lymphoma patients and controls (*p* = 0.07).

**Fig. 3:**
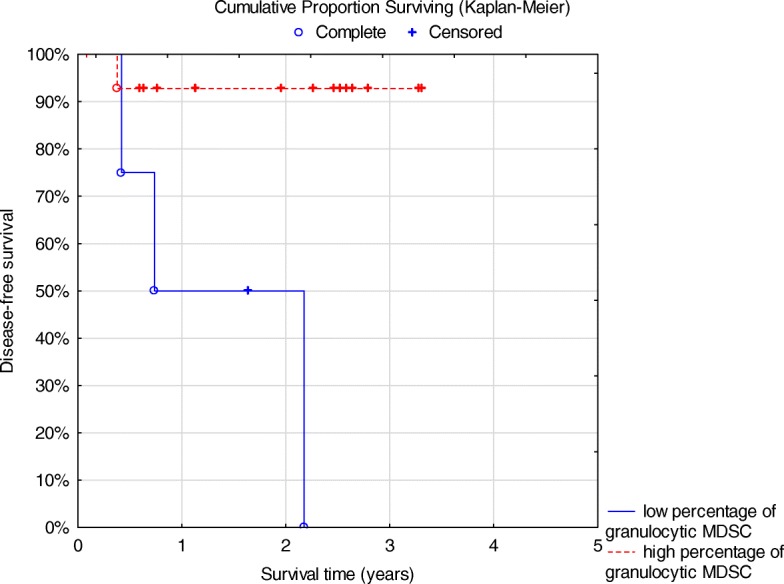
Disease-free survival for classical Hodgkin lymphoma patients with high granulocytic MDSC (*n* = 15) vs low (*n* = 4) *p* = 0.04 log rank test

**Fig. 4:**
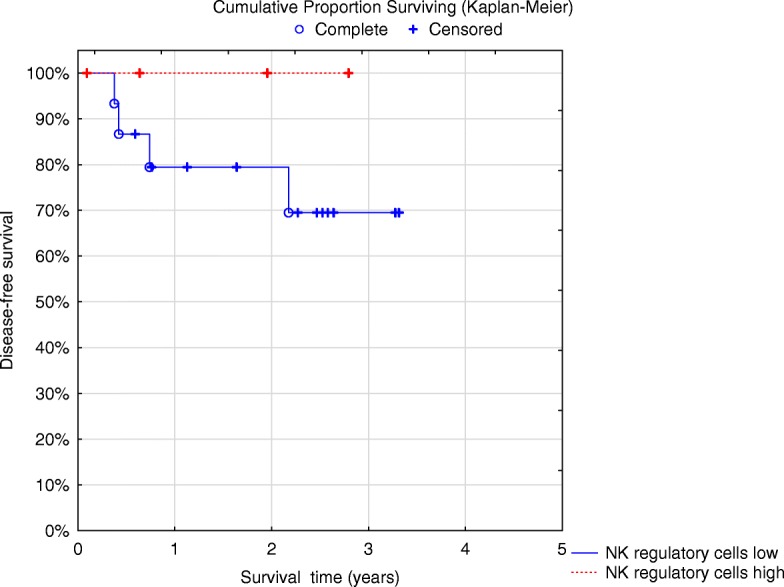
Disease-free survival of classic Hodgkin lymphoma patients (*n* = 15) low vs high percentage of NK regulatory cells (*n* = 4) *p* = 0.8 log rank test. Patients with high percentage of NK regulatory cells are all alive (red line) at 100%

There were no statistical differences between the percentages of different immune cells when comparing cHL and HGBCL patients (data not shown) and no significant statistical differences regarding DFS except for granulocytic MDSCs in cHL (*p* = 0.04) (Fig. 3). Furthermore, it appeared that cHL patients with higher percentages of regulatory NK cells had a superior DFS since all patients were alive (Fig. 4) but this was not statistically significant (*p* = 0.8) and patient numbers were small.

One patient with advanced stage 4B of cHL nodular sclerosis (syncytial variant containing numerous tumor cells), with an IPS of 4, was treated with BEACOPP and is still alive with no signs of recurrence. At diagnosis, he had a favorably normal percentage of monocytes, high percentage of T cells and high percentages of regulatory NK cells and granulocytic MDSCs. In contrast, the three patients who died of cHL had higher amounts of monocytes and all three had low (below median) amounts of regulatory NK cells and low granulocytic MDSCs. One patient who relapsed in cHL also had a low percentage of regulatory NK cells and all had an IPS of 3.

For the HGBCL patients, three died of lymphoma and two of other malignancies with no sign of lymphoma. For those who died of lymphoma 2/3 had international prognostic index (IPI) 4 and a high percentage of monocytes and lower than median of regulatory NK cells (one patient was not analyzed).

## Discussion

Our patients with newly diagnosed lymphomas showed an altered profile of immune cells in the peripheral blood. The percentage of T cells was decreased which has been observed by others but there were no significant differences regarding the T regulatory cells [9, 23].

In this study we show a significantly lower percentage of regulatory NK cells in newly diagnosed lymphoma patients, both cHL and HGBCL compared to healthy blood donors. In a recently published study, the subset of CD56^bright/^CD16^dim/−^ regulatory NK cells was found to express PD1^high^ which might further explain why cHL patients are more prone to respond to PD1-blockade [11]. In our study the CD56^bright/^CD16^dim/−^ regulatory NK cells were decreased in cHL patients and in contrast appeared to be associated with a better outcome, which has not been reported previously. We were, however, unable to analyze PD-1 on NK cells since this marker was not included in our tube of NK cell markers.

The amount of regulatory NK cells may decrease with increasing age [24, 25] and it can thus not be ruled out that increased age may explain parts of these results, although we did not find any differences in relation to age. However, there was also a significant decrease for the cHL patients who constitute a younger population, more comparable to the healthy blood donors. There was also a tendency towards an improved outcome for the cHL patients with increased regulatory NK cells, a finding that needs to be confirmed in larger cohorts of cHL patients. This better outcome for the cHL patients may be explained by an increased NK cell-mediated cytotoxicity towards T regulatory cells, a finding that needs to be further explored and can indicate a possible dual role for these cells. It would also be of interest to evaluate whether such regulatory NK cells can show cytotoxicity against B-cell-derived tumor cells.

Granulocytic MDSCs were identified in the granulocytic compartment and the definitions of PMN MDSCs in humans have been diverse and difficult to clearly characterize [17, 18, 26, 27]. However, granulocytic MDSCs or PMN MDSCs may be considered to be activated, low-density granulocytes collected in the mononuclear layer after density gradient separation. These neutrophils have immunosuppressive properties, negatively regulating host immune response in cancer patients but with an immunophenotype that cannot be separated from neutrophils [28]. In our study we defined cells in the granulocytic compartment positive for CD45^+^/CD11b^+^/CD33^+^/CD15^+^/CD14^−^/HLA-DR^−^ as granulocytic MDSCs. An attempt was made to use CD11b, CD13 and CD16 as markers of mature granulocytes and CD16^dim/−^ for immature granulocytes based on granulocyte maturation patterns known in the bone marrow [29]. However, the results did not differ significantly if all cells with the immunophenotype: CD45^+^/CD11b^+^/CD33^+^/CD15^+^/CD14^−^/HLA-DR^−^ in the granulocytic compartment were used compared to those with a slightly decreased expression of CD16. Thus, a more reliable definition of MDSCs should be related to density properties and function since it appears as though these cells lack a specific immunophenotype. In addition, it might not be ruled out that the ficoll separation process may actually affect the expression of some markers which has been reported for CD16 [30], or that some cell populations are lost which hampers the evaluation of the MDSCs. Indeed, ficoll separation was developed for sorting the mononuclear cell population. Furthermore, cryopreservation has been discussed to affect the function of MDSCs and a decrease in the total number might be observed, but most previous studies have, however, been performed on cryopreserved cells [18, 27, 28]. In addition, it is also difficult to report the absolute numbers for comparisons between the samples. In some cases there will be a larger amount of living cells included and thus the absolute number of cells will therefore not give a justified comparison where rather the frequencis/percentages of cells would be preferable. The samples are analysed after ficoll separation of mononuclear cells why a comparison to for instance white blood cell counts value also would be inappropriate.

MDSCs have been studied in several different cancer types and are mostly increased at diagnosis [18, 26, 31] which we also observed for the lymphoma patients. In contrast to other studies, where an increase in PMN MDSCs has been associated with a worse outcome for cancer patients [31–33], our cHL patients who had an increase in PMN MDSCs had a significantly superior DFS, but our patient material was small. CHL patients had more MDSCs, both monocytic and granulocytic, compared to HGBCL and healthy blood donors. This has been observed in some previous studies [31, 34] where lymphoma patients had higher amounts of MDSCs. Nevertheless, for malignancies that derive from the immune system itself, immune regulatory cells may not only affect the anti-tumor immune responses but they may affect the tumor cells as well. There is clearly a need to better understand immune regulation in patients with lymphomas. However, independently of a possible interaction of regulatory cells and the tumor, it may be necessary to take into consideration the different subsets of immune regulatory cells when decisions are made on immunotherapies, since some patients may be more prone to respond while some cHL patients may be identified who may benefit most from blocking PD-1/PD-L1 interactions.

There was a male predominance in our lymphoma cohort both for cHL and HGBCL patients which is somewhat higher than we previously have observed in other population-based cohorts [35, 36]. Patient samples were collected prospectively and we cannot fully explain this difference. Clinical characteristics like age, stage and IPS were almost comparable to our previous studies of cHL although it appeared as if the HGBCL patients were somewhat less advanced regarding both stage and IPI, which may influence the results since we did not find any survival differences regarding this patient group. Nevertheless, the HGBCL patients did have a DFS comparable to our previous data. Three-year DFS of 73% for the cHL patients is inferior compared to other studies and may be explained by the fact that some of the cHL patients were older in this rather limited cohort. The primary aim was not, however, to make comparisons regarding the clinical characteristics and outcome. In addition, the survival analyses of the immune cell subsets may be influenced by suboptimal treatment like bendamustin only in some case.

## Conclusions

In conclusion, the composition of different subtypes of immune cells in the peripheral blood of newly diagnosed lymphoma patients differs from healthy blood donors although not to a statistically significant extent regarding all investigated subtypes. However, there were no major differences regarding the lymphoma diagnoses; cHL or HGBCL. T-cells and regulatory NK cells were significantly decreased in lymphoma patients, whereas granulocytic MDSCs were increased.

In cHL patients, an increased percentage of granulocytic MDSCs was associated with a better DFS and an increased percentage of regulatory NK cells had a tendency to be associated with a superior outcome for cHL patients which has not been shown previously, although the number of patients was low. Nonetheless, further studies are needed in order to confirm our findings and explore the favorable immune signature in these patients.

## Additional files

Additional file 1: Gating strategies. The different gating strategies for the different immune cells are presented. (PPTX 624 kb)

Additional file 2: ROC curves. The ROC curves for the different immune cell are presented. Reciever operating curves (ROC) to determine optimal cutoffs for CD3+ T cells, T-regulatory cells, Monocytes, NK cells, NK regulatory cells, Monocytic MDSC, Granulocytic MDSC and Granulocytes regarding disease-free survival are presented. (PDF 51 kb)

## Abbreviations

ABVD: Doxorubicine, bleomycin, vinblastine, dacarbazine
ADF: Alive disease free
AVD: Doxorubicine, vinblastine, dacarbazine
AWD: Alive with disease
BEACOPP: Bleomycin, etoposide, doxorubicin, cyclophosphamide, vincristine, procarbazine, prednisone
CHL: Classical Hodgkin Lymphoma
DFS: Disease-free survival
DLBCL: Diffuse large B-cell lymphoma
DWD: Dead with disease
HGBCL: High grade B-cell lymphoma
IPI: International prognostic index, stage according to Ann Arbor classification
IPS: International prognostic score
MDSC: Myeloid derived suppressor cells
PD: Progressive disease
PD1: Programmed death receptor 1
PMN: Polymorphonuclear
R-CHOP: Rituximab, cyclophosphamide, doxorubicine, vincristine, prednisone
RT: Radiotherapy
