# Supplementary material for: Altered profile of immune regulatory cells in the peripheral blood of lymphoma patients
Source: BMC Cancer. 2019 Apr 5;19:316. doi: 10.1186/s12885-019-5529-0 (PMC6449984; doi:10.1186/s12885-019-5529-0)
Supplement: Supplementary file 1 — Gating strategies. The different gating strategies for the different immune cells are presented. (PPTX 624 kb) [file 12885_2019_5529_MOESM1_ESM.pptx]

## Slide 1
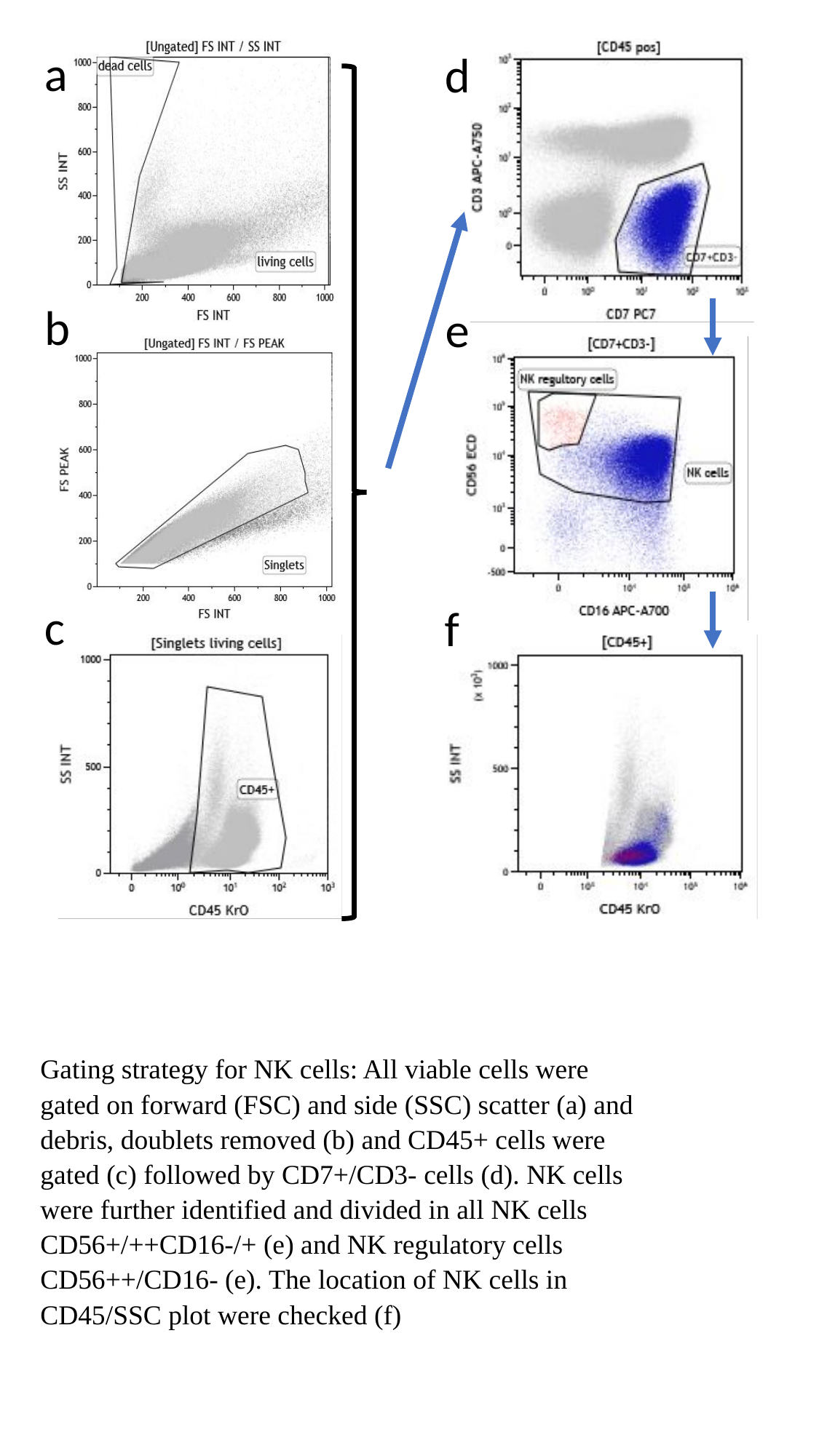

a
d
b
e
c
f
Gating strategy for NK cells: All viable cells were gated on forward (FSC) and side (SSC) scatter (a) and debris, doublets removed (b) and CD45+ cells were gated (c) followed by CD7+/CD3- cells (d). NK cells were further identified and divided in all NK cells CD56+/++CD16-/+ (e) and NK regulatory cells CD56++/CD16- (e). The location of NK cells in CD45/SSC plot were checked (f)

## Slide 2
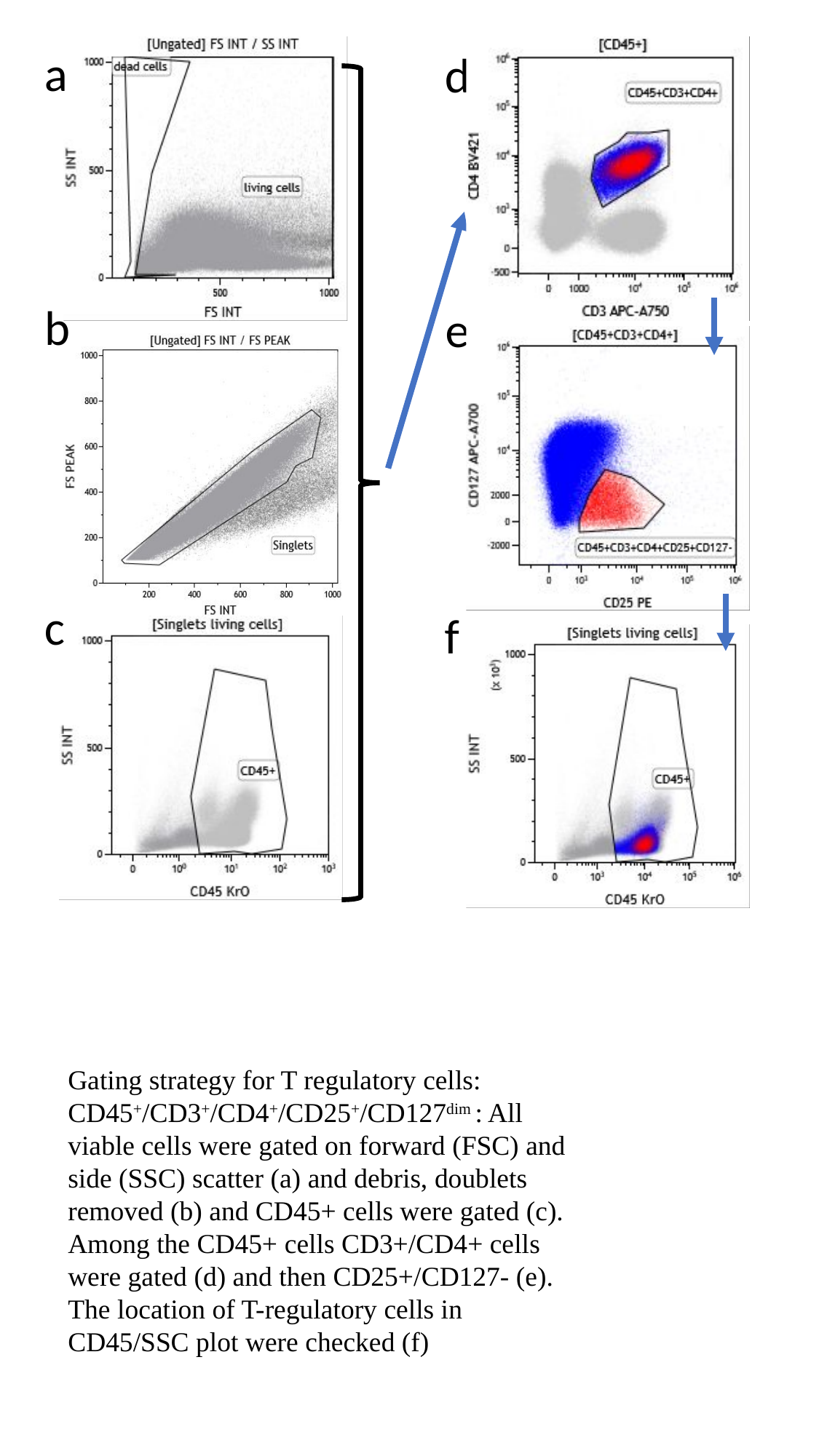

a
d
b
e
c
f
Gating strategy for T regulatory cells: CD45+/CD3+/CD4+/CD25+/CD127dim : All viable cells were gated on forward (FSC) and side (SSC) scatter (a) and debris, doublets removed (b) and CD45+ cells were gated (c). Among the CD45+ cells CD3+/CD4+ cells were gated (d) and then CD25+/CD127- (e). The location of T-regulatory cells in CD45/SSC plot were checked (f)

## Slide 3
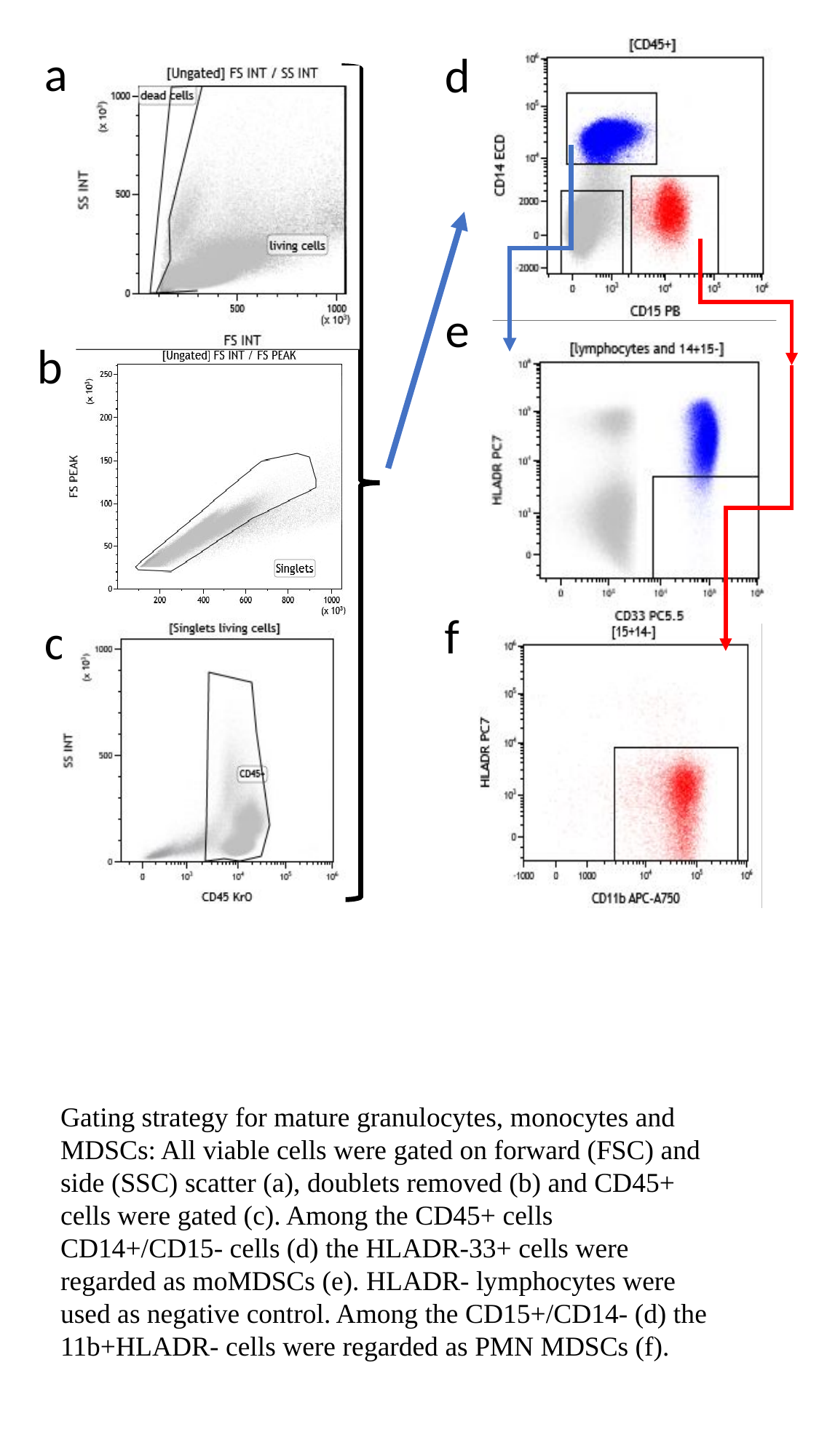

a
d
e
b
f
c
Gating strategy for mature granulocytes, monocytes and MDSCs: All viable cells were gated on forward (FSC) and side (SSC) scatter (a), doublets removed (b) and CD45+ cells were gated (c). Among the CD45+ cells CD14+/CD15- cells (d) the HLADR-33+ cells were regarded as moMDSCs (e). HLADR- lymphocytes were used as negative control. Among the CD15+/CD14- (d) the 11b+HLADR- cells were regarded as PMN MDSCs (f).
