# Supplementary material for: Altered profile of immune regulatory cells in the peripheral blood of lymphoma patients
Source: BMC Cancer. 2019 Apr 5;19:316. doi: 10.1186/s12885-019-5529-0 (PMC6449984; doi:10.1186/s12885-019-5529-0)
Supplement: Supplementary file 2 — ROC curves. The ROC curves for the different immune cell are presented. Reciever operating curves (ROC) to determine optimal cutoffs for CD3+ T cells, T-regulatory cells, Monocytes, NK cells, NK regulatory cells, Monocytic MDSC, Granulocytic MDSC and Granulocytes regarding disease-free survival are presented. (PDF 51 kb) [file 12885_2019_5529_MOESM2_ESM.pdf]

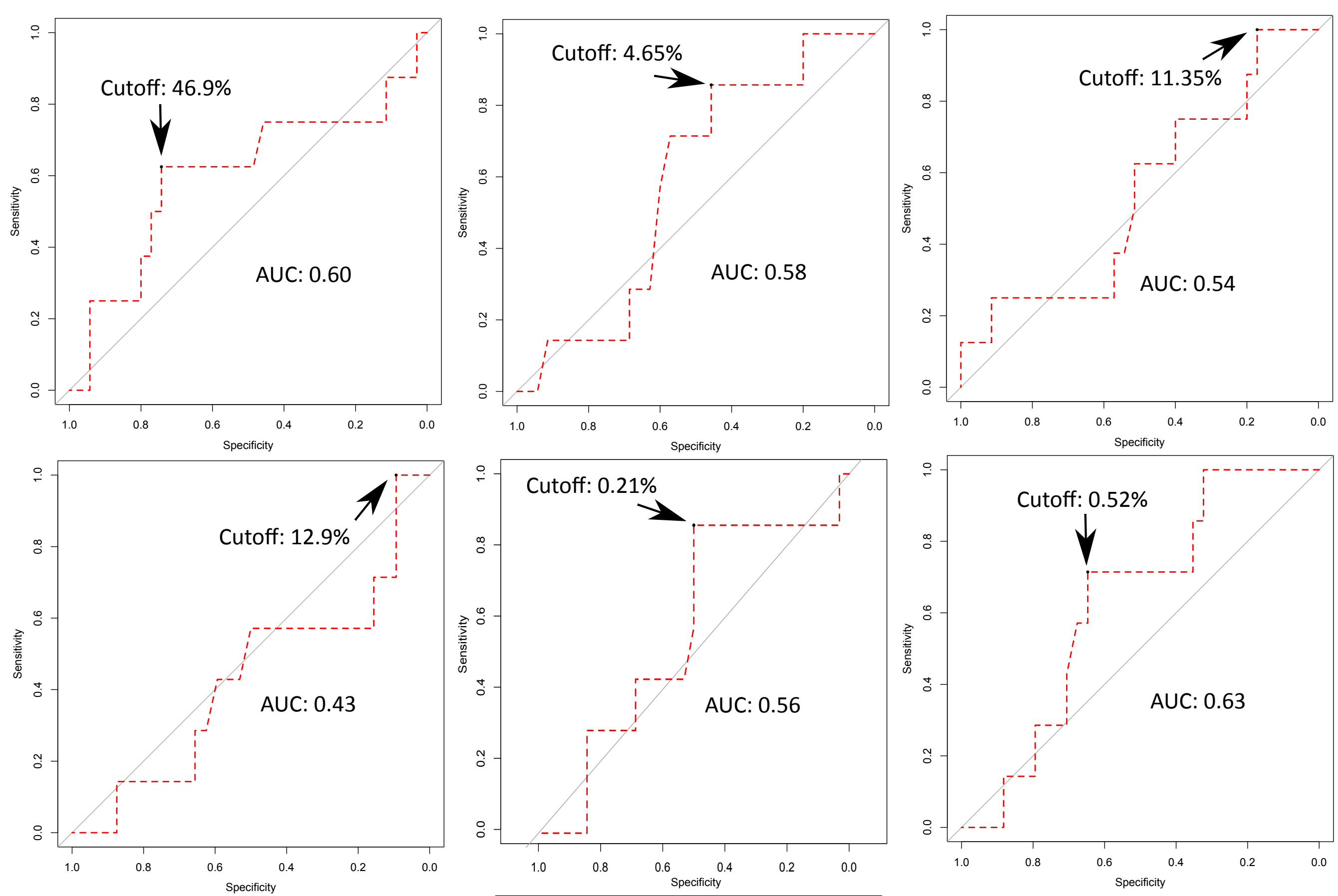

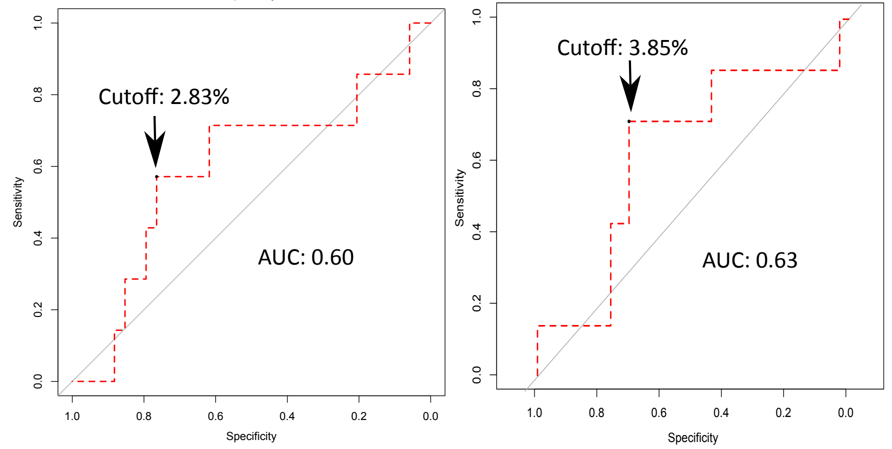

Receiver operating characteristic curves to determine optimal cutoffs for CD3+ T cells (A), T-regulatory cells (B), Monocytes (C), NK cells (D), NK regulatory cells (E), Monocytic MDSC (F), Granulocytic MDSC (G) and Granulocytes (H) regarding disease-free survival.

Abbreviations: AUC, Area Under Curve; NK, Natural Killer; MDSC, Myeloid Derived Suppressor Cells.
